# *CRK41* Modulates Microtubule Depolymerization in Response to Salt Stress in *Arabidopsis*

**DOI:** 10.3390/plants12061285

**Published:** 2023-03-12

**Authors:** Sa Zhou, Qiuling Luo, Zhiyan Nie, Changhui Wang, Wenkang Zhu, Yingxiang Hong, Jun Zhao, Baolei Pei, Wenjian Ma

**Affiliations:** 1 Key Laboratory of Industrial Fermentation Microbiology of the Ministry of Education, College of Biotechnology, Tianjin University of Science and Technology, Tianjin 300457, China; zhousa@tust.edu.cn (S.Z.);; 2 State Key Laboratory of Plant Physiology and Biochemistry, College of Biological Sciences, China Agricultural University, Beijing 100193, China; 3 College of Life Science and Food Engineering, Huaiyin Institute of Technology, Huai’an 223003, China; 4 Qilu Institute of Technology, Jinan 250200, China

**Keywords:** *CRK41*, MPK3, MPK6, microtubule depolymerization, salt stress

## Abstract

The pivotal role of cysteine-rich receptor-like kinases (CRKs) in modulating growth, development, and responses to stress has been widely acknowledged in *Arabidopsis*. However, the function and regulation of *CRK41* has remained unclear. In this study, we demonstrate that *CRK41* is critical for modulating microtubule depolymerization in response to salt stress. The *crk41* mutant exhibited increased tolerance, while overexpression of *CRK41* led to hypersensitivity to salt. Further analysis revealed that CRK41 interacts directly with the MAP kinase3 (MPK3), but not with MPK6. Inactivation of either MPK3 or MPK6 could abrogate the salt tolerance of the *crk41* mutant. Upon NaCl treatment, microtubule depolymerization was heightened in the *crk41* mutant, yet alleviated in the *crk41mpk3* and *crk41mpk6* double mutants, indicating that *CRK41* suppresses MAPK-mediated microtubule depolymerizations. Collectively, these results reveal that *CRK41* plays a crucial role in regulating microtubule depolymerization triggered by salt stress through coordination with MPK3/MPK6 signalling pathways, which are key factors in maintaining microtubule stability and conferring salt stress resistance in plants.

## 1. Introduction

Plants have developed a wide array of receptor-like protein kinases (RLKs) in response to different environmental and internal stimuli. RLKs are a type of serine–threonine protein kinase that consists of three distinct parts: an extracellular domain, a transmembrane helix, and a conserved intracellular protein kinase catalytic domain [1]. RLKs can convert extracellular signals into intracellular signals and activate downstream signal pathways to regulate a number of defensive and developmental processes. Cysteine-rich receptor-like kinases (CRKs) are a large family of RLKs, containing 46 members, which are thought to be linked to the control of plant growth and development, as well as the reactions to different environmental stimuli [1,2].

In *Arabidopsis thaliana*, CRK family members are crucial in regulating the responses to biotic and abiotic stressors. When confronted with biotic stress and pathogen associated molecular pattern (PAMP) treatments, CRKs exhibit a rapid and drastic increase in the expression level. *CRK4*, *CRK6*, *CRK13*, *CRK20*, *CRK28*, *CRK29*, *CRK36*, and *CRK45* are resistant to bacteria such as *Pseudomonas syringae* Pv. *Tomato* DC3000 [3,4,5], and *CRK36* also contributes to responsive to necrotrophic fungal pathogen *Alternaria brassicicola* in *Arabidopsis* [6]. ALS1, a typical CRK in rice, is involved in Salicylic acid (SA), jasmonate, and NH1-mediated defense responses [7], while NH1-mediated immunological responses are mediated by *CRK6* and *CRK10* [8]. *TaCRK1* in wheat mediates the defense response against *Rhizoctonia cerealis* through ABA signaling pathway [9], and *HvCRK1* in barley can regulate the defense response against saprophytic fungal barley powdery mildew [10]. *GbCRK18* affected the resistance of *Gossypium barbadense* to *Verticillium dahlia* [11]. 

Research has also demonstrated that CRKs play a role in controlling abiotic stress tolerance. Overexpression of *CRK4*, *CRK5*, *CRK19*, and *CRK45* enhances ABA sensitivity of stomatal movement and drought tolerance in early seedlings [12]. *CRK5* affects the release of reactive oxygen species (ROS) in response to ultraviolet light [13], and *CRK45* acts as a positive regulator in seed germination and seedling growth in response to salt stress [14]. Additionally, *CRK6*, *CRK7*, *CRK8*, *CRK10*, and *CRK15* show increased transcript levels after exposure to O_3_ and work together to enable a suitable response to oxidative stress induced by O_3_ (which induces extracellular ROS production) in *Arabidopsis thaliana* [1,15]. Salt stress also leads to an increase in ROS levels, resulting in oxidative stress. Excessive production of ROS causes serious oxidative damage, while appropriate production of ROS acts as a defense signal to improve stress tolerance [16]. Moreover, *TaCRK41* was involved in response to salt and drought stress in wheat [17]. Study has revealed that *CRK41* has a suppressive impact on salt tolerance in *Arabidopsis* [18]. Consequently, it is evident that CRKs are essential for the reaction to biotic and abiotic stresses, but their functional studies focus mainly on resistance to biotic stress, which is only rarely reported on abiotic stress resistance in *Arabidopsis thaliana*. However, there is limited understanding about the molecular regulatory processes of *CRK41* in plants when exposed to salt stress. 

Microtubules, which are highly dynamic polymers that can depolymerize and polymerize, are involved in various cellular processes [19]. The polymerization–depolymerization dynamics of microtubules are influenced by a range of developmental processes, as well as environmental stimuli and stress conditions [19,20,21]. Microtubules have been identified as playing a role in the amplification of signals, as well as being integral components in the processing of stress signals, which are closely related to salt stress [20]. Studies also have demonstrated that salt stress can alter the organization of cortical microtubules [22]. Moreover, microtubule stability and dynamic affect salt tolerance in *Arabidopsis*. Research has found that the application of paclitaxel, a stabilizer of microtubules, decreases the seedlings survival rate of *Arabidopsis* during salt stress, whereas propyzamide and oryzalin, which are known to disrupt microtubules, increase the survival rate [23]. Thus, rapid microtubule depolymerization is advantageous in helping plants to tolerate salt stress [20,21,22,23].

Furthermore, MAPK signalling pathways, initially thought to be responsible for the regulation of microtubule organization and dynamic instability, have been found to be involved in the growth, development and stress response of plants [24,25,26,27]. MAPKs can be activated when *Arabidopsis* is exposed to biotic and abiotic stresses [28]. MAPKs phosphorylate microtubule-associated proteins (MAPs), which can assist the coordinated action of the assembly and disassembly dynamics of microtubule and modulate the affinity of proteins to microtubule surface [29,30,31]. Studies have revealed a connection among three *Arabidopsis* MAPKs, MPK4, MPK6, and MPK13, and microtubule organization and dynamics [27]. These MAPKs have been observed to interact with MAPs, with MPK4 and MPK13 interacting with MAP65-1, MAP65-2, and MAP65-3, and MPK6 interacting with MAP65-1 [27]. Additionally, MAP65-1 has been determined to be a protein that can be effectively phosphorylated by MPK4 and MPK6 [25,32]. MPK3 can be colocalized with MAP65-1, as well as microtubules [23,33]. Investigation has shown that phosphorylation of MAP65-1 reduces its capability to form microtubule bundles in vitro, which could consequently initiate the depolymerization of microtubules [23,34]. 

In general, salt stress signals can be transduced into cells by plasma membrane proteins, which result in dynamic modifications of microtubules. To date, little is known about the function of plasma membrane protein CRK41 during salt stress; it was further explored in this study by using wild type, *crk41* mutant, and overexpressed lines of *CRK41*. Moreover, the mechanism of *CRK41*’s regulated responses to salt stress was also elucidated. 

## 2. Results

### 2.1. The Basic Characteristics of CRK41

**Figure 1:**
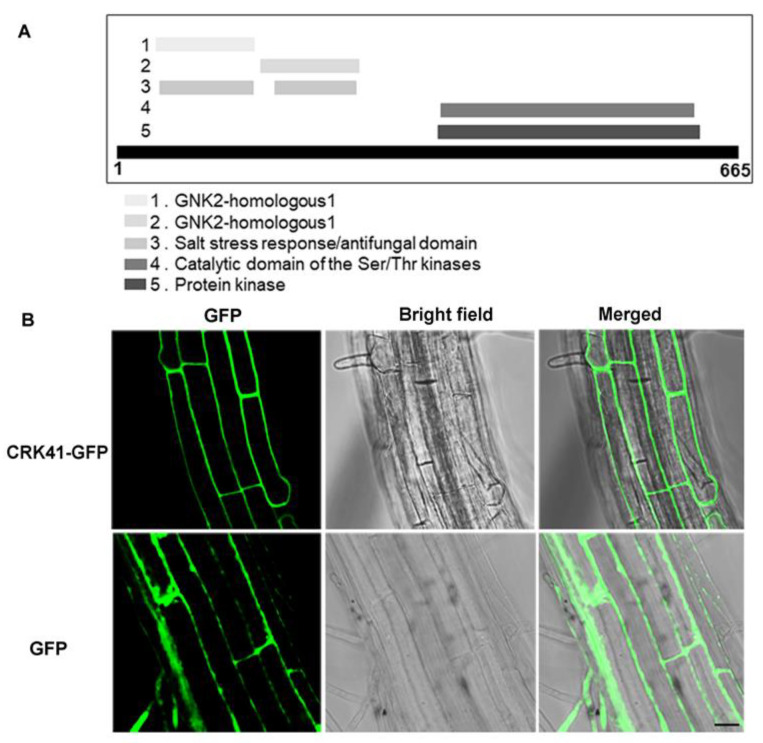
The basic characteristics of CRK41. (**A**) The functional domain composition analysis of CRK41 was from the website: https://www.ebi.ac.uk/interpro/protein/UniProt/O23081/ (accessed on 8 November 2022). (**B**) The localization of CRK41 in roots cells (bar = 20 μm). Three replicates of similar results are obtained in the experiments.

We firstly analyzed the functional domain composition of CRK41 to determine the fundamental characteristics of *CRK41*. The result revealed that CRK41 contained the DUF26 domain, protein kinase domain, and transmembrane domain, which was made up of 665 amino acids (aa) (Figure 1A). To assess its cellular localization, we transformed wild type with a *35S:CRK41-GFP* vector to create *35S:CRK41-GFP* lines. Results indicated that CRK41 was found to be situated on the plasma membrane in the root cells of *Arabidopsis* (Figure 1B). Therefore, we speculate that the function of *CRK41* is primarily to sense the changes in environmental signals. 

### 2.2. crk41 Mutant Increased Salt Tolerance

**Figure 2:**
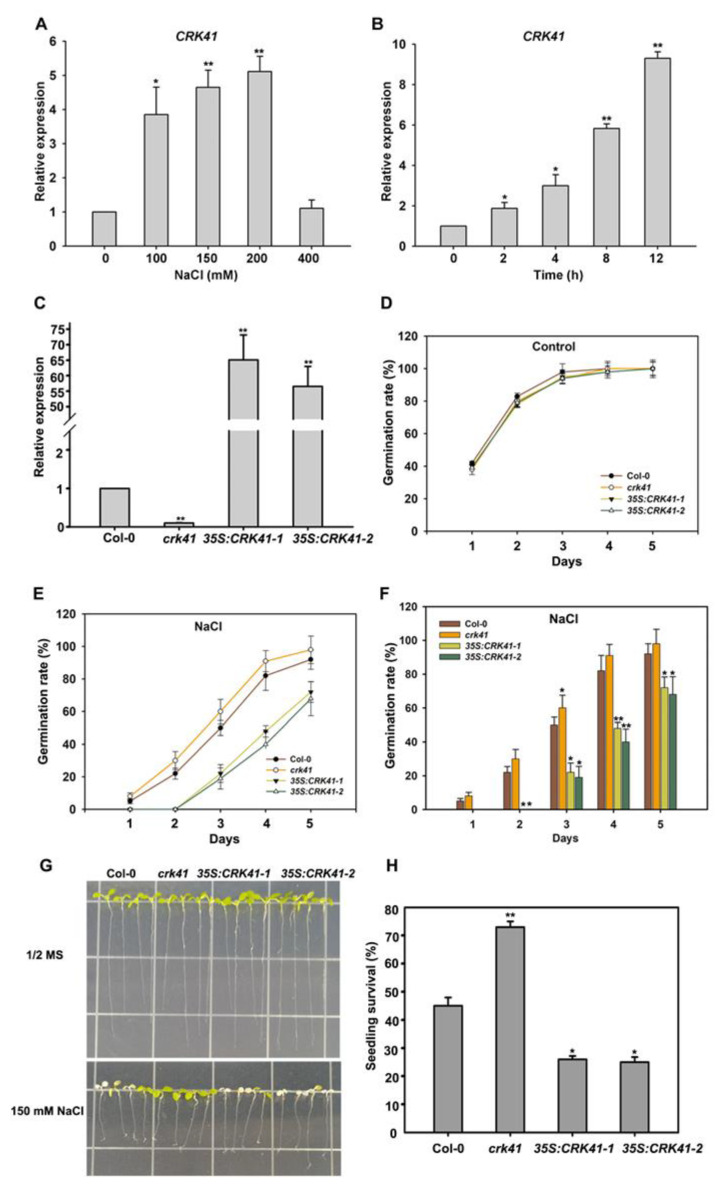
*CRK41* has an inhibitory effect on salt stress tolerance. (**A**,**B**) The relative expression level of *CRK41* was determined after NaCl treatment for different concentrations (0, 100, 150, and 200 mM) (**A**) and times (0, 2, 4, 8, and 12 h) (**B**). The standard deviation (SD) is represented by the error bars; *n* = 3. * *p* < 0.05; ** *p* < 0.01 vs. Control. (**C**) The relative expression level of *CRK41* in Col-0, *crk41* mutant, *35S:CRK41-1*, and *35S:CRK41-2* under normal condition. SD is represented by the error bars; *n* = 3. ** *p* < 0.01 vs. wild type. (**D**–**F**) The seed germination rate of four lines, including *crk41* mutant, *35S:CRK41-1*, and *35S:CRK41-2*, was assessed on 1/2 MS medium without (**D**) or with 120 mM NaCl (**E**,**F**). Observations were made daily post-transfer to assess germination rate. SD is represented by the error bars; *n* = 3 (80 seeds were measured in per genotype of each replicate). * *p* < 0.05; ** *p* < 0.01 vs. wild type. (**G**,**H**) An analysis of salt sensitivity (**G**) and seedling survival rate (**H**) were conducted on wild type, *crk41* mutant, *35S: CRK41-1*, and *35S: CRK41-2* after 150 mM NaCl treatment. SD is represented by the error bars; *n* = 3 (at least 60 seeds were measured in per genotype of each replicate). * *p* < 0.05; ** *p* < 0.01 vs. wild type.

An examination of the role of *CRK41* in salt stress was carried out through the measurement of its gene expression level in Col-0 following NaCl treatment. Real-time PCR analysis showed an increase expression level of *CRK41* upon exposure to different times and concentrations of NaCl (Figure 2A,B). The transcript level of *CRK41* was also detected after different concentrations of mannitol. Osmotic stress caused by mannitol had no effect on the expression level of *CRK41* (Figure S1). This indicates that *CRK41* can be induced by salt stress, and the response of *CRK41* is specific to NaCl.

Furthermore, to investigate the influence of *CRK41* in salt stress tolerance, two *CRK41* overexpression lines, *35S: CRK41-1* and *35S: CRK41-2*, were produced by introducing the *35S: CRK41* vector into Col-0. Then, the gene expression level of *CRK41* in the *crk41* mutants, *35S: CRK41-1* and *35S: CRK41-2*, was determined. Quantitative PCR results showed a notable increase in the expression level of *CRK41* in *35S: CRK41-1* and *35S: CRK41-2*, whereas *CRK41* was barely expressed in the *crk41* mutant. The results indicated that the *crk41* mutant and *CRK41* overexpression lines *35S: CRK41-1* and *35S: CRK41-2* could be used for phenotypic analysis. Then, the seed germination of Col-0, *crk41*, *35S: CRK41-1*, and *35S: CRK41-2* was observed after treatment with NaCl. The seeds of the indicated genotypes were sown on 1/2 MS plates with 120 mM NaCl, the seeds germination was observed in 5 days, and the seed germination rate was counted. Without NaCl, the genotype of the seed had no impact on the rate of germination (Figure 2D). The germination rate of *crk41* was greater than that of Col-0 when the medium contained 120 mM NaCl, whereas the germination rate of *35S: CRK41-1* and *35S: CRK41-2* was decreased compared with Col-0 (Figure 2E,F). This indicates that the *crk41* mutant had greater salt resistance. 

In order to provide further evidence that *CRK41* was implicated in salt stress, a phenotypic assessment of Col-0, *crk41*, *35S: CRK41-1*, and *35S: CRK41-2* after NaCl treatment was conducted. When grown without NaCl, the lengths of roots and leaves of Col-0, *crk41* mutant, *35S: CRK41-1*, and *35S: CRK41-2* showed no significant difference. However, after transferring 6-day-old seedlings to a plate with 150 mM NaCl, the leaves of *35S: CRK41-1* and *35S: CRK41-2* were drastically discolored and bleached compared with Col-0 after 3 d NaCl treatment. Interestingly, the *crk41* mutant showed lesser bleaching than the wild type (Figure 2G). We further evaluated the survival of seedlings by examining leaf bleaching during salt stress, a sign of seedling death. If two cotyledons both had turned white, the seedling was determined to be dead, while all other seedlings were classified as living. The survival rate was calculated on the basis of the ratio of living seedlings to the total number of seedlings. The survival rate of wild type seedlings was substantially lower than that of *crk41* mutant and higher than that of *35S: CRK41-1* and *35S: CRK41-2* after NaCl treatment (Figure 2H). 

Trypan blue staining was used to assess the effect of *CRK41* on leaf cell-death-related phenotypes. In comparison with Col-0, the *crk41* mutant exhibited fewer cells stained by trypan blue after salt treatment, indicating reduced cell death (Figure S2). Additionally, electrolyte leakage was measured in Col-0, *crk41* mutant, and *CRK41* overexpression lines after treatment with medium containing 150 mM NaCl. The results revealed that the plasma membrane of the *CRK41* overexpression lines had more severe damage than that of Col-0, while the *crk41* mutant had only mild damage (Figure S3).

To confirm that the salt-tolerant phenotype in *crk41* mutant was a result of the disruption of the *CRK41* gene, the *crk41* complemented lines *CRK41/crk41-5* and *CRK41/crk41-6* were utilized, which restored the salt-sensitive phenotype and were similar to that of the wild type (Figure S4).

Our findings suggested that *CRK41* was a vital factor for the resistance to salt stress. Results from seed germination, phenotypic observation, leaf cell death, plasma membrane damage, and gene expression study indicated that *CRK41* has an inhibitory effect on salt stress tolerance. 

### 2.3. CRK41 Has Been Identified as a Factor in the Alteration of Microtubule Depolymerization When Exposed to Salt Stress

**Figure 3:**
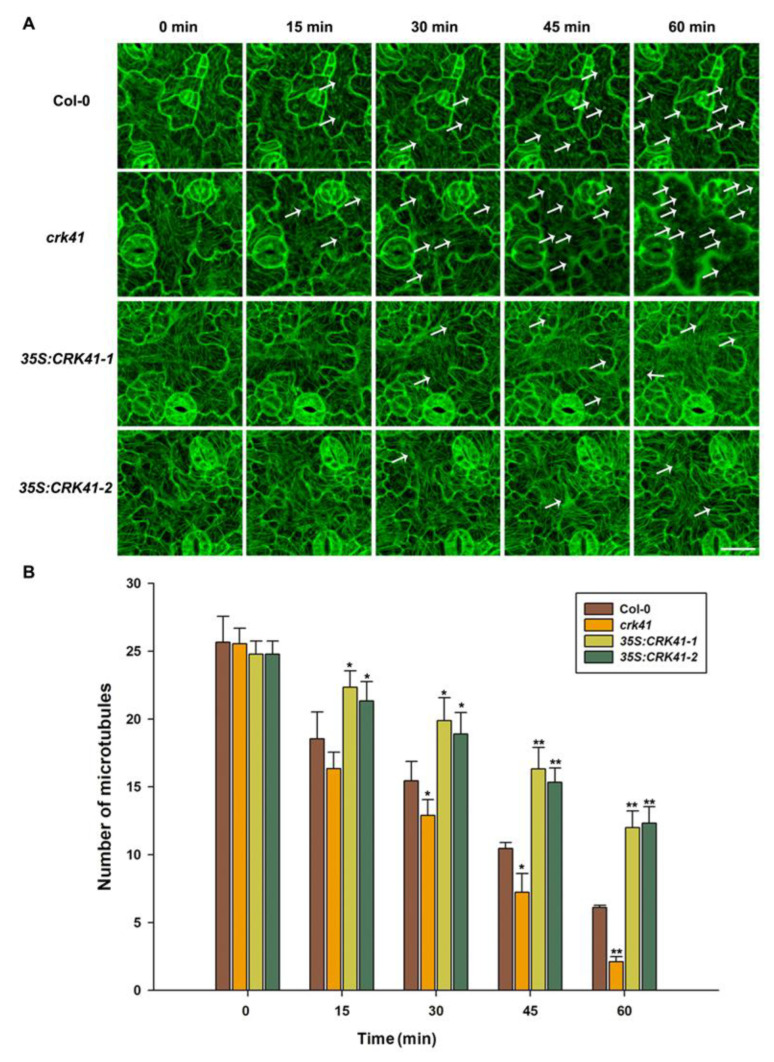
*CRK41* has been identified as a regulator of cortical microtubule depolymerization when exposed to 150 mM NaCl. (**A**) Analysis of the microtubule modifications caused by 150 mM NaCl of Col-0, *crk41* mutant, *35S:CRK41-1*, and *35S:CRK41-2* was conducted through a series of sequential images (scale bar = 20 μm). The arrows indicated representative microtubule depolymerization. (**B**) Image tool software was utilized to quantify cortical microtubules of Col-0, *crk41* mutant, *35S:CRK41-1*, and *35S:CRK41-2*, with a minimum of 18 cells from 3 samples being analyzed. SD is represented by the error bars. * *p* < 0.05; ** *p* < 0.01 vs. wild type in the same condition.

Research has revealed that microtubule depolymerization is a significant factor in the signaling pathways of both biotic and abiotic stress [21,22,27,34,35]. Moreover, microtubule dynamic instability is required for salt stress tolerance [35,36,37]. A rapid depolymerization of microtubules is beneficial to plant survival under salt stress [21,36]. Therefore, we determined whether *CRK41* was a crucial factor for controlling the depolymerization of microtubules during salt stress. The relationship between *CRK41* and microtubule disassembly during salt stress was investigated using Col-0, *crk41* mutant, *35S: CRK41-1*, and *35S: CRK41-2*. The microtubules were visualized directly in Col-0, *crk41* mutant, *35S: CRK41-1*, and *35S: CRK41-2* seedlings after 150 mM NaCl treatment at various times (0, 15, 30, 45, and 60 min). No substantial difference was observed in terms of microtubule organization and density prior to salt application. After treatment with 150 mM NaCl, the cortical microtubules were depolymerized, and the level of depolymerization varied among Col-0, *crk41* mutant, and *CRK41* overexpression lines. Wild type cotyledon pavement cells displayed a dramatic depolymerization in cortical microtubule with extended treatment duration; in contrast, the *35S:CRK41* overexpression line showed a weaker depolymerization in cortical microtubule (Figure 3). Moreover, the *crk41* mutant demonstrated a more extreme and rapid depolymerization of cortical microtubules compared with wild type (Figure 3). The results suggest that *CRK41* appears to be a key factor for controlling microtubule depolymerization in response to salt stress.

### 2.4. CRK41 Interacts with MPK3 

MAPKs were initially perceived as a mechanism to control the arrangement and activity of microtubules; however, over time, it was determined that MAPKs were responsible for mediating defence reactions to biotic and abiotic stresses in plants [28]. Studies in *Arabidopsis* have revealed that MAPKs MPK3/MPK6 and MPK4 phosphorylate HSFA4A and SOS1, two heat shock factors, are found to enhance salt tolerance [38]. Furthermore, evidence suggested that MAPK cascades played a critical role in the downstream pathways of RLKs [39]. Previous research has also demonstrated that the YODA-MKK4/5-MPK3/MPK6 signaling pathway is regulated by ERECTA (ER), a RLK that controls localized cell proliferation in plants [32,40]. Consequently, we aimed to determine whether CRK41 physically interacts with MAPKs signaling in plants.

**Figure 4:**
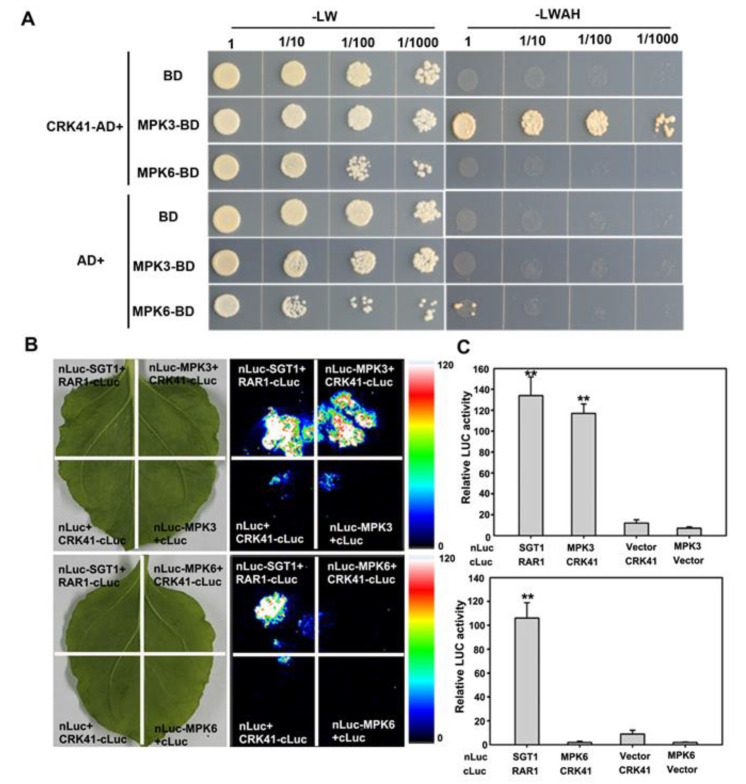
CRK41 interacts with MPK3. (**A**,**B**) Evidence of the interaction among CRK41 and MPK3 and MPK6 was obtained via a yeast two-hybrid assay (**A**) and a LCI assay (**B**). SGT1-nLUC and RAR1-cLUC served as a positive control, while MPK3-nLUC + GUS-cLUC, MPK6-nLUC + GUS-cLUC, CRK41-cLUC + GUS-nLUC, and CRK41-cLUC + GUS-nLUC served as negative controls in (**B**). (**C**) The relative luminescence of the sample in (**B**) was measured. SD is represented by the error bars, *n* = 3. ** *p* < 0.01 vs. MPK3-nLUC + GUS-cLUC/MPK6-nLUC + GUS-cLUC group.

In order to determine the interaction between CRK41 and MPK3/MPK6, a yeast two-hybrid assay was carried out. It was observed that CRK41 interacted with MPK3 in yeast (Figure 4A). A Luciferase complementation imaging (LCI) assay was then conducted on *Nicotiana tabacum* leaves to investigate the interaction between CRK41 and MPK3/MPK6 in plants. This revealed that CRK41 interacted with MPK3 in plants (Figure 4B). Nevertheless, neither the yeast two-hybrid assay nor the LCI assay detected any interaction between CRK41 and MPK6 (Figure 4).

### 2.5. CRK41 Is Responsible for the Regulation of MPK3 and MKP6, Both of Which Are Integral in Salt Stress Tolerance

**Figure 5:**
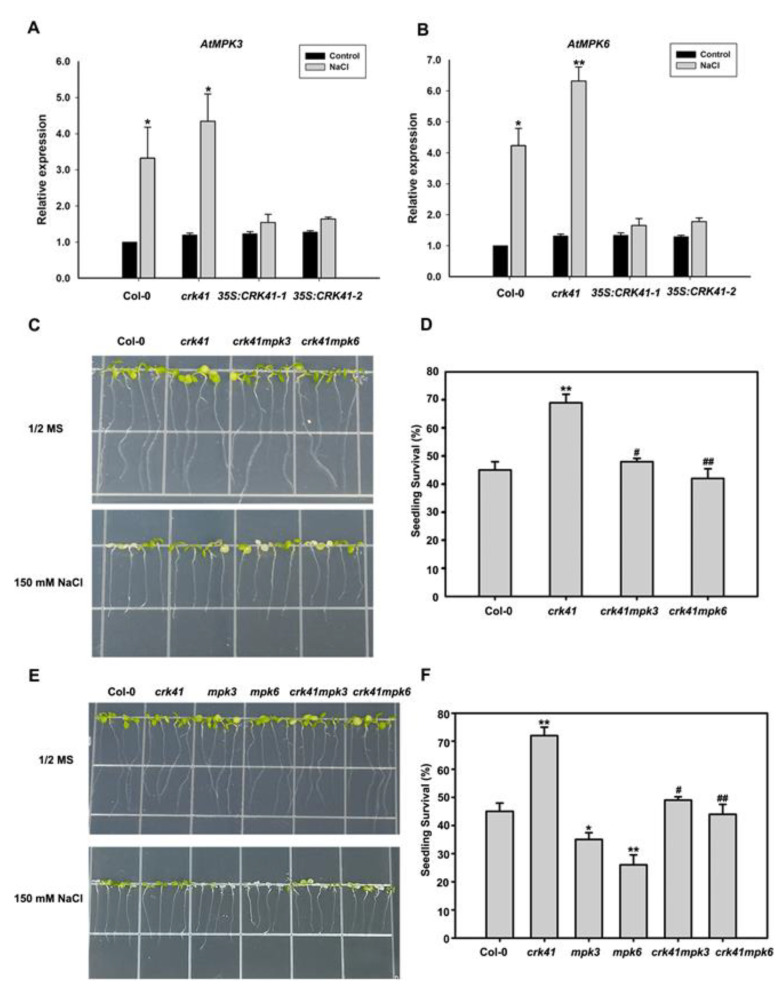
*MPK3* and *MKP6* are required in *crk41* mutant during salt stress. (**A**,**B**) The relative expression levels of *MPK3* and *MPK6* were determined using real-time PCR analysis after ddH2O or 150 mM NaCl treatment for 12 h in wild type. The seedlings treated with ddH2O were used as control. SD is represented by the error bars; *n* = 3. * *p* < 0.05; ** *p* < 0.01 vs. Control in the same genotype. (**C**,**E**) The growth phenotypes of the seedlings in Col-0, *crk41*, *crk41mpk3*, *crk41mpk6*, *mpk3*, and *mpk6* mutants after 150 mM NaCl treatments. (**D**,**F**) Seedling survival rates for Col-0, *crk41*, *crk41mpk3*, *crk41mpk6*, *mpk3*, and *mpk6* mutants. The survival rate of seedlings was then measured after NaCl treatment for 5 d. SD is represented by the error bars; *n* = 3 (at least 60 seeds were measured in per genotype of each replicate). * *p* < 0.05; ** *p* < 0.01 vs. wild type; ^#^ *p* < 0.05; ^##^ *p* < 0.01 vs. *crk41* mutant.

To further explore whether *CRK41* impacted *MPK3* and *MPK6* when exposed to salt stress, real-time PCR was used to measure *MPK3* and *MPK6* expression level in Col-0, *crk41* mutant, *35S: CRK41-1*, and *35S: CRK41-2* seedlings. Following NaCl treatment, the transcript levels of *MPK3* and *MPK6* were significantly elevated in Col-0 and *crk41* mutant, with the expression level of *MPK3* and *MPK6* in *crk41* mutant exhibiting a greater increase than that of Col-0. Conversely, no increase in the transcript levels of *MPK3* and *MPK6* was observed in *35S: CRK41-1* or *35S: CRK41-2* (Figure 5A,B).

In order to investigate whether the altered gene expression of *MPK3* and *MPK6* was the cause for the salt tolerance of *crk41*, *crk41mpk3* and *crk41mpk6* were generated by crossing *crk41* with *mpk3* and *mpk6*. Six-day-old seedlings of wild type, *mpk3*, *mpk6*, *crk41mpk3*, and *crk41mpk6* mutants were moved to plates containing either no NaCl or 150 mM NaCl. After 3 days of the NaCl treatment, phenotypic analyses revealed that the *mpk3* and *mpk6* mutants had much more severe chlorosis and bleaching of their leaves than the wild type, while the leaves in *crk41* mutant showed less bleaching than the wild type (Figure 5E). However, the *crk41mpk3* and *crk41mpk6* mutants did not demonstrate any remarkable variations in comparison with the wild type (Figure 5C,E). Moreover, the survival rate *mpk3* and *mpk6* was notably reduced when compared with wild type, whereas the survival rate of *crk41mpk3* and *crk41mpk6* mutants was comparable to that of wild type (Figure 5D,F).

Results showed that *crk41* mutant had a greater resistance to salt stress, while deletion of *MPK3* and *MPK6* genes in the *crk41* mutant restored its salt tolerance phenotype (Figure 5C–F). These results demonstrate that *MPK3* and *MPK6* are required to regulate salt stress tolerance caused by the mutation of *CRK41*.

### 2.6. MPK3 and MKP6 Are Essential Components in CRK41-Modulating Salt-Stress-Induced Microtubule Depolymerization

To confirm the molecular mechanism of *MPK3* and *MPK6* in reaction to NaCl, we determined whether *MPK3* and *MPK6* affected microtubule depolymerization. The effects of NaCl treatment on microtubule depolymerization were observed in Col-0, *mpk3*, and *mpk6* mutant seedlings. Prior to the application of salt, no observable disparities were seen in the microtubule arrangement and concentrations of the *mpk3* and *mpk6* mutants in comparison with those of the wild type. The depolymerization of cortical microtubules was less pronounced in the *mpk6* mutant than in Col-0, while the *mpk3* mutant had a slightly reduced depolymerization compared with the wild type (Figure S5). Thus, it appears that *MPK3* and *MPK6* are both implicated in the control of microtubules depolymerization when exposed to salt stress, with *MPK6* playing a particularly significant role. 

**Figure 6:**
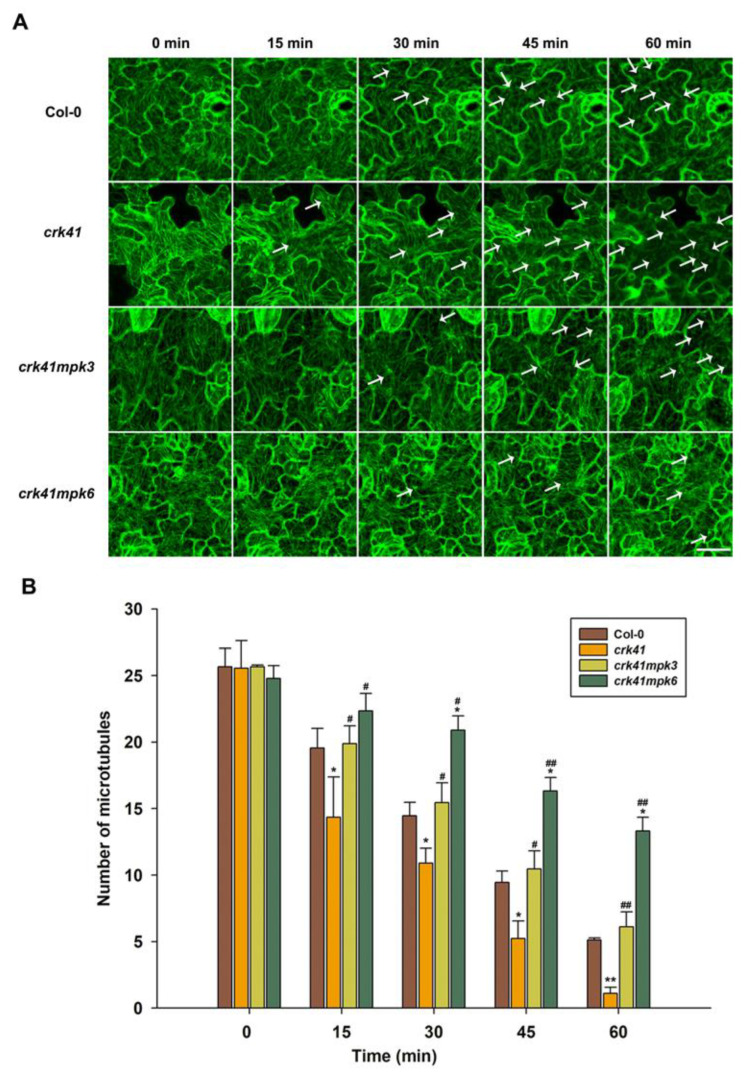
*MPK3* and *MPK6* are essential components in *CRK41*-modulating microtubule depolymerization induced by NaCl. (**A**) Analysis of the microtubule modifications in Col-0, *crk41*, *crk41mpk3*, and *crk41mpk6* was conducted induced by 150 mM NaCl through a series of sequential images (scale bar = 20 μm). The arrows indicated representative microtubule depolymerization. (**B**) Image tool software was utilized to quantify cortical microtubules of Col-0, *crk41*, *crk41mpk3*, and *crk41mpk6*, with a minimum of 18 cells from 3 samples being analyzed. SD is represented by the error bars. * *p* < 0.05; ** *p* < 0.01 vs. wild type in the same condition, ^#^ *p* < 0.05; ^##^ *p* < 0.01 vs. *crk41* mutant in the same condition.

In addition, to further determine whether *MPK3* and *MPK6* modulated microtubule depolymerization in *crk41* mutant in response to salt stress, the microtubule dynamics of seedlings in wild type, *mpk3*, *mpk6*, *crk41mpk3*, and *crk41mpk6* mutants were investigated after NaCl treatment. With NaCl treatment, microtubule depolymerization occurred, and microtubule density decreased in wild type, *crk41mpk3*, and *crk41mpk6* mutants. The *crk41mpk3* showed a similar level of microtubule depolymerization to wild type; however, the *crk41mpk6* mutant exhibited a weakened depolymerization (Figure 6).

The findings indicated that both *MPK3* and *MPK6*, especially *MPK6*, promote microtubule depolymerization of the *crk41* mutant, and deletion of *MPK3* and *MPK6* inhibits depolymerization of *crk41* mutant microtubules. These data revealed that *MPK3* and *MPK6* are connected to regulating the reaction to salt stress by depolymerizing microtubules in the *crk41* mutant.

Our data indicate that *CRK41* modulates the expression levels of *MPK3* and *MPK6* when exposed to salt stress and appears to be an inhibitory regulator of these genes. It is clear that *MPK3* and *MPK6* are vital in the microtubule depolymerization of the *crk41* mutant in the presence of salt stress. 

## 3. Discussion

CRKs have been found to be essential in controlling growth and development, in addition to the modulation of defence or immune responses and abiotic stress [1,14,41,42,43]. Recent studies have demonstrated that the elimination of *CRK41* enhances the salt tolerance of *Arabidopsis*, while its overexpression increases the plant’s sensitivity to salt stress [18]. However, there have been no genetic data to support the role that *CRK41* plays in salt stress tolerance. Our research demonstrated that that a loss-of-function mutation in the *Arabidopsis* gene *CRK41* results in increased expression of *MPK3* and *MPK6*, as well as alterations in depolymerization rate of microtubules. 

Previous study indicated that a novel wheat CRK gene, *CRK41*, has an effect on the germination of *Arabidopsis thaliana* seeds when exposed to osmotic stress, and *TaCRK41* product is deposited in the cytoplasm [17]. A recent study revealed that CRK41 was co-localized with endocytic membrane markers of FM4−64 in *Arabidopsis* root cells as analyzed by genetic transformation systems, indicating that CRK41 is localized in the plasma membrane [18]. Consistent with previous studies, our research also demonstrated that CRK41 product is deposited in plasma membrane and contains transmembrane domain (Figure 1). We speculate that the role of *CRK41* in salt stress tolerance is primarily to sense salt-sensitive signals and transfer extracellular signals into intracellular signals.

In eukaryotic cells, the cytoskeleton is constantly undergoing disassembly and rearrangement due to the triggering of a variety of signaling pathways. The cortical microtubules, which are integral components of the cytoskeleton, perform a range of roles in cell development and plants’ response to various abiotic stress, particularly salt stress [35,36,37]. Several studies have indicated that depolymerization and rearrangement of cortical microtubules are strategies employed by plants to boost their salt tolerance, and the rapid and global microtubule depolymerization promoted plant resistance to salt stress [35,36,37,44]. Recent studies have shown that the changes in microtubule dynamics can also be beneficial for suspension cells to adapt to salt stress [45]. Moreover, we previously demonstrated that H2Bub1 plays a role as a facilitator in plant salt tolerance by stimulating the depolymerization of cortical microtubules [35]. We also determined that *MAP65-1* modulates and accelerates the degradation of microtubules in reaction to salt stress [23]. Our research revealed that absence of *CRK41* caused a significant depolymerization in the microtubules, while *CRK41* overexpression caused a milder depolymerization (Figure 3). These results imply that *CRK41* appears to be able to influence the microtubule depolymerization when exposed salt stress. Results from the molecular genetic analysis revealed that the *crk41* mutant had an increased capacity to withstand salt stress, as indicated by its loss-of-function. In contrast, *CRK41* overexpression lines, *35S:CRK41-1* and *35S:CRK41-2*, were more hypersensitive to salt than wild type (Figure 2). Moreover, studies have shown that *CRK41* also took part in salt-induced oxidative stress. The *crk41* mutant displayed reduced ROS accumulation when compared with Col-0 after NaCl treatment, whereas overexpression lines of *CRK41* resulted in increased levels [18]. Our results determined that the membrane damage after oxidative stress was more severe in *CRK41* overexpression lines, while the plasma membrane of the *crk41* mutant was only slightly affected (Figure S3). These results suggested that *CRK41* was involved in multiple aspects of salt stress response. Among them, microtubule dynamics are a fundamental element of plant cells and are necessary for the successful implementation of cellular processes to help plants better adapt and tolerate salt stress. The microtubule depolymerization phenotype of *crk41* mutant and *CRK41* overexpression lines in this study further demonstrated that plants enhance salt tolerance by managing the depolymerization and rearrangement of cortical microtubules [44,45]. 

Plant resilience to stress tolerance conditions is contingent upon a multitude of factors, among which MAPK signalling is a key regulatory mechanism [46]. MAPK signals, particularly those reliant on MPK3/MPK6, are essential for the response to a variety of biotic and abiotic stressors in plants [47,48,49]. MKK5-MPK3/MPK6 can be activated after NaCl treatment and mediates to regulate the expression of iron superoxide dismutase genes to adapt to salt stress in *Arabidopsis*. Genetic evidence showed that MKK5-RNAi plants presented a salt-sensitive phenotype [50]. *MKK5^DD^* triggered MPK3 and MPK6 activity, which enhanced salt tolerance [51]. Exposing plants to high salinity activates MPK3/MPK6, which phosphorylate and destabilize downstream factors, eventually promoting salt tolerance in *Arabidopsis* [51,52,53]. Our preceding study found that activation of MPK3 and MPK6 is triggered in response to salt stress and is a key factor in salt stress tolerance [23,35]. However, there were few reports about the association between CRK41 and MPK3/MPK6 in salt stress. Our findings indicated that CRK41 was able to interact with MPK3 in vitro and in vivo (Figure 4), and *CRK41* participated in the regulation of the expression of *MPK3* and *MPK6* (Figure 5A,B). Molecular genetic analysis showed that loss functions of *mpk3* and *mpk6* mutants were hypersensitive to NaCl treatment, compared with wild type (Figure 5E). These results indicated that *CRK41* may mediate salt stress responses through MPK3 and MPK6 signalling module in *Arabidopsis*.

Increasing evidence has connected MAPK activation to conditional and developmental microtubule rearrangements. *MPK4* and *MPK6* are involved in regulating cytoskeletal in *Arabidopsis*, and MPK6 colocalizes with microtubules [26,54]. *MPK4* and *MPK18* have been identified as regulating the stability of microtubules, as loss-of-function *mpk18-1* mutant has more enduring and drug-resistant cortical microtubules. Moreover, the *mpk4* mutant exhibits a more severe phenotype [25,55]. Our research revealed that *MPK3* and *MPK6*, especially *MPK6*, affected microtubule dynamics when exposed to salt stress (Figure S5). Genetic analysis showed that loss of *mpk3* and *mpk6* mutants alleviated the rapid microtubule depolymerization of the *crk41* mutant (Figure 6). As shown in Figure 4, CRK41 can interact with MPK3. Our previous research revealed that MPK3 and MPK6 interact with MAP65-1, a microtubule binding protein that influences microtubule stability [23]. We hypothesized that *CRK41* could have an effect on MAP65-1 through MPK3, thus impacting microtubule stability. Thus, *MPK3* and *MPK6* play essential roles in regulating microtubule depolymerization during salt stress response of *CRK41.* Salt-sensitive phenotypes were also analyzed in *crk41mpk3* and *crk41mpk6* double mutants. *crk41mpk3* and *crk41mpk6* mutants restored the salt tolerance phenotype of *crk41*, and the salt tolerance of *crk41mpk3* was comparable to the wild type, while *crk41mpk6* was slightly more responsive to NaCl treatment compared with the wild type (Figure 5). The phenotypic results were consistent with those of microtubule depolymerization, and further proved that salt tolerance requires microtubule dynamics. 

## 4. Materials and Methods

### 4.1. Plant Materials

Wild type (Col-0), *crk41* (SALK_056649.45.60.x) [18], *35S:CRK41-1*, *35S:CRK41-2*, *CRK41/crk41-5*, *CRK41/crk41-6*, *mpk3* (SALK_100651), *mpk6*(SALK_062471) [56], *crk41mpk3*, and *crk41mpk6* mutants (all with a Col-0 background) were used in this study. The T-DNA insertion mutants *crk41*, *mpk3*, and *mpk6* were acquired from State Key Laboratory of Plant Physiology and Biochemistry *Arabidopsis* seeds platform. The *crk41*, *mpk3*, and *mpk6* mutants were identified as homozygous mutants by PCR. The *mpk3* and *mpk6* mutants were confirmed by activity analysis of MPK3 and MPK6 [35]. The *35S:CRK41-1* and *35S:CRK41-2* were generated by transforming the wild type with *35S:CRK41* vector. The *crk41* complemented lines *CRK41/crk41-5* and *CRK41/crk41-6* were generated by transforming the *crk41* mutant with *35S:CRK41* vector. The *crk41mpk3* and *crk41mpk6* mutants were generated by crossing *crk41* with *mpk3* and *mpk6.*

Seeds were subjected to a sterilization process using 0.2% sodium hypochlorite prior to sowing, and 1/2 MS medium with 1% (*w*/*v*) sucrose and 0.8% (*w*/*v*) agar was used as the growing medium in Petri dishes. The plants were grown under a 16 h light/8 h dark photoperiod and 70% relative humidity in a growth cabinet.

### 4.2. Subcellular Localization

For subcellular localization analyses of *CRK41*, full-length cDNA of *CRK41* was incorporated into *35S:CRK41-GFP* vector. The homozygous transgenic lines of *35S:CRK41-GFP* were produced by introducing Col-0 with *35S:CRK41-GFP* vector. The deposition of CRK41 was examined using a laser confocal microscope Zeiss LSM710 (Carl Zeiss Micro Imaging GmbH, Oberkochen, Germany).

### 4.3. Salt Sensitivity Assay

For the seed germination assay, 80 seeds of wild type, *crk41*, *35S:CRK41-1*, and *35S:CRK41-2* were planted on 1/2 MS medium with or without 120 mM NaCl for 5 d. Germination (defined as the apparent emergence of radicles) was monitored at the various times. 

Additionally, seeds of the wild type, *crk41*, *35S:CRK41-1*, *35S:CRK41-2*, *mpk3*, *mpk6*, *crk41mpk3*, and *crk41mpk6* mutants were sown on plates for 6 days. Then, at least 60 seedlings of each genotype were transplanted to plates containing either 150 mM NaCl or no NaCl for 3 d to analyze the phenotypic. The percentage of seedlings surviving was determined after treatment for 5 days.

### 4.4. Detection of Cell Death in Leaves

The cell death in leaves was quantified by staining with trypan blue, following the protocol as described by Bowling et al. [57]. Three independent biological replicates were performed.

### 4.5. Ion Leakage Assays

Seven-day-old seedlings were transferred from 1/2 MS medium to 1/2 MS medium containing 150 mM NaCl. After 0, 2, 4, 8, and 12 h, seedlings were removed from plates, washed with deionized water, and placed in tubes containing 5 mL of deionized water. Then, ion leakage was measured and calculated as described previously [35].

### 4.6. Quantitative Real-Time PCR

*Arabidopsis* total RNA was isolated with Trizol reagent (Invitrogen, Carlsbad, CA, USA) and subjected to DNaseI (Invitrogen) treatment. The reverse-transcribed cDNA was synthesized with PrimeScript Reverse Transcription kit (TaKaRa, Dalian, China). Real-time PCR analysis was conducted using cDNA as the template, with SYBR Premix Ex Taq Kit (Takara) and an ABI 7500 Real-Time PCR machine (Applied Biosystem, Foster City, CA, USA). Primers for real-time PCR to detect *CRK41* (AT4G00970), *MPK3* (AT3G45640), and *MPK6* (AT2G43790) were provided in Supplementary Table S1, with ACTIN2 as the internal control. 

### 4.7. Yeast Two-Hybrid Assays

Yeast culture, transformation, and analysis were conducted using methods previously documented in [58]. The full-length cDNAs of *MPK3* and *MPK6* were inserted into pGBKT7 (Clontech, Mountain View, CA, USA) as the bait vector, while *CRK41* full-length cDNA was inserted into pGADT7 (Clontech) as the prey vector. Primers in Supplementary Table S1 were used to amplify *CRK41*, *MPK3*, and *MPK6* cDNAs. Co-transformation of pGBKT7-MPK3/pGBKT7-MPK6 and pGADT7-CRK41 into AH109 competent cells was conducted, and the cells were grown in SD-Trp-Leu medium. Negative controls were picked using the empty plasmids of pGBKT7 and pGADT7. Positive clones that appeared were picked and inoculated onto SD-Trp-Leu-His-Ade plates. Three independent biological replicates were performed.

### 4.8. LCI Assays

Amplification and recombination of the *MPK3* and *MPK6* was performed to generate the nLUC plasmid, while *CRK41* were amplified and recombined into cLUC plasmid. *Agrobacterium tumefaciens* strain GV3101 was utilized to transiently transfect the ligated nLuc-MPK3/nLuc-MPK6 and CRK41-cLuc into 5-week-old *N.benthamiana*, and then leaves were collected after 2–3 days. Primers in Supplementary Table S1 were used to amplify of *CRK41-*, *MPK3*-, and *MPK6*-coding sequences. A positive control was picked using the plasmid of nLuc-SGT1 and RAR1-cLuc. Negative controls were picked using the empty plasmids of nLUC and cLUC. The LCI assay and relative LUC activity was performed [59].

### 4.9. Observation of Microtubule Organization under Salt Stress

Wild type, *crk41*, *35S:CRK41-1*, *35S:CRK41-2*, *mpk3*, *mpk6*, *crk41mpk3*, and *crk41mpk6* mutants stably expressing GFP–tubulin were used for this assay. The leaves of 7-day-old seedlings were incubated with 150 mM NaCl, then the disassembly and reorganization of the cortical microtubules the organization and disassembly of the cortical microtubules was observed over time using a Zeiss LSM 710 microscope (Carl Zeiss Micro Imaging GmbH). The procedure and protocol were described by Shi et al. [60]. Microtubules were analyzed in z stacks of images at 1.5 μm intervals. Zeiss LSM 710 software was used to acquire fluorescent images of microtubules, which were then converted to TIFF format and further edited in Adobe Photoshop 5.0. The number of microtubules was calculated, as described previously [61]. Microtubule number was counted along a fixed line (~50 μm) perpendicular to the direction of most cortical microtubules in the cell using ImageJ software (NIH, Bethesda, MD, USA).

### 4.10. Statistical Analysis

Statistical analysis was conducted using Student’s *t*-test and one-way ANOVA in SPSS 26.0. A statistical significance was noted if the *p*-value was less than 0.05, which was denoted by * and ^#^ (*,^#^ = *p* < 0.05, **,^##^ = *p* < 0.01).

## 5. Conclusions

**Figure 7:**
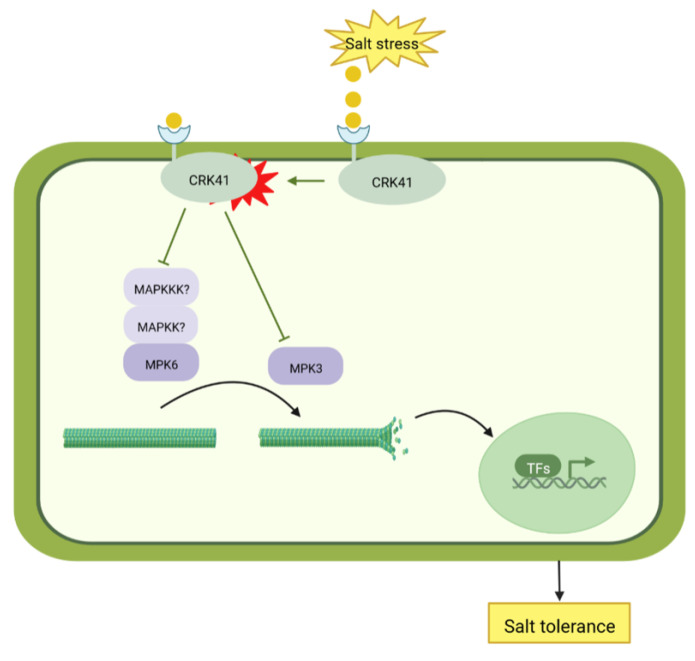
Model of *CRK41*-mediated salt tolerance.

The current research indicates that *CRK41* has a negative effect on *Arabidopsis* salt tolerance due to its influence on microtubule dynamic instability. MPK3/MPK6 are essential for the reaction of *crk41* mutant in salt stress. CRK41 is associated with MPK3, not MPK6. *MPK3* and *MPK6*, especially *MPK6*, are essential for the microtubule depolymerization, which is necessary for salt tolerance in the *crk41* mutant (Figure 7). Microtubule dynamics can also be used as a signal, transmitting salt signals into cell nucleus, and influencing the expression of salt-related genes. Eventually, *Arabidopsis* can adapt to salt stress.

## Data Availability

The data presented in this study are available within the article.

## Supplementary Materials

The following supporting information can be downloaded at: https://www.mdpi.com/article/10.3390/plants12061285/s1, Table S1: Primers used in this work; Figure S1: The relative expression level of *CRK41* was determined after different concentrations of mannitol treatment; Figure S2: Cell death induced by different concentrations of NaCl in leaves of Col-0 and *crk41* mutant; Figure S3: Ion leakage in seedlings of Col-0, *crk41* mutant, *35S:CRK41-1*, and *35S:CRK41-2* treated with NaCl; Figure S4: The salt-sensitive phenotype could be restored in the *crk41* complemented lines; Figure S5: *MPK3* and *MKP6* have been identified as regulators of cortical microtubule depolymerization when exposed to salt stress.
