# Supplementary material for: CRK41 Modulates Microtubule Depolymerization in Response to Salt Stress in Arabidopsis
Source: Plants (Basel). 2023 Mar 12;12(6):1285. doi: 10.3390/plants12061285 (PMC10051889; doi:10.3390/plants12061285)
Supplement: Supplementary file 1 [file plants-12-01285-s001.zip › plants-2155057-supplementary.pdf]

Table S1. Primers used in this work.

Primers used for quantitative real-time PCR

AGI	Gene	Primer_forward (5'–3')	Primer_reverse (5'–3')
AT3G45640	*MPK3*	GATTTGTGAT-TTCGGTCTTGC	TTTAC-CAGGGAACAAAGGCT
AT2G43790	*MPK6*	CATTATCCGAAGAACATT-GCC	TACTT-GGTTTCAAATCCCTGTG
AT4G00970	*CRK41*	CCAAATCATCCCAATTCTC C	TAGATTCACTGCGGCTT-GAG
AT3G18780	*ACTIN2*	TACAGTGTCTG-GATCGGTGGTT	CGGCCTTGGA-GATCCACAT

Primers used for PCR fragments

AGI	Gene	Primer_forward (5'–3')	Primer_reverse (5'–3')
AT3G45640	*MPK3*	ATGAACAC-CGGCGGTGGCCAAT	CTAACCGTATGTT-GGATTGAG
AT2G43790	*MPK6*	ATGGAC-GGTGGTTCAGGTCAACC	CTATTGCTGATATTCTG-GATTG
AT4G00970	*CRK41*	ATGACTAGTTCTT-GTTCTTTGTC	ACGAGCATCGAACTCGG-TAATT

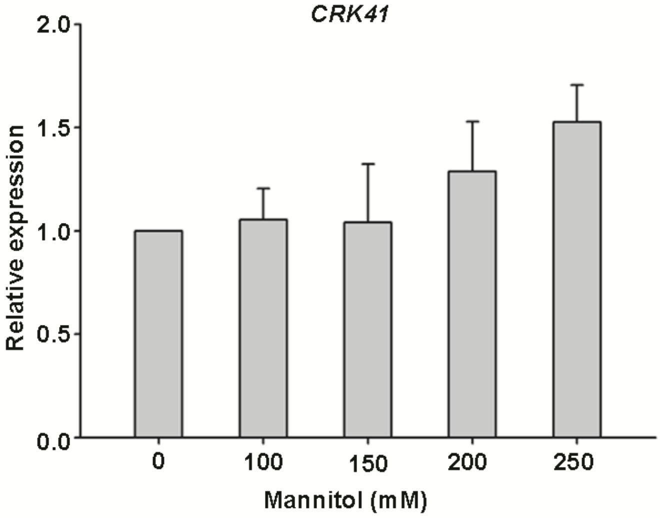

Figure S1. The relative expression level of *CRK41* was determined after different concentrations of mannitol treatment. SD is represented by the error bars; *n* = 3.

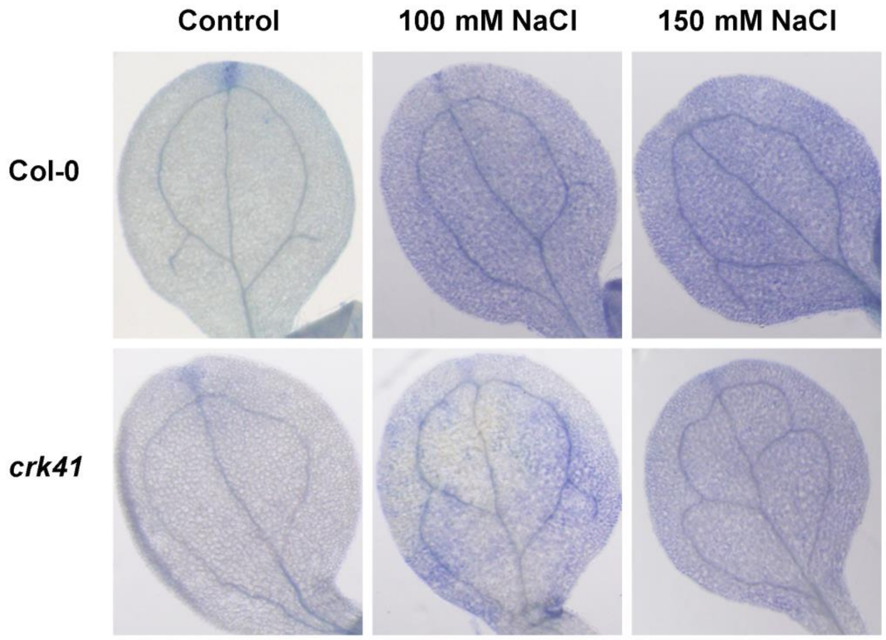

Figure S2. Cell death induced by different concentrations of NaCl in leaves of Col-0 and *crk41* mutant. 7-day-old plants were treated with 100 mM and 150 mM NaCl for 18 h and stained with trypan blue. The leaves treated with ddH_2O were used as control.

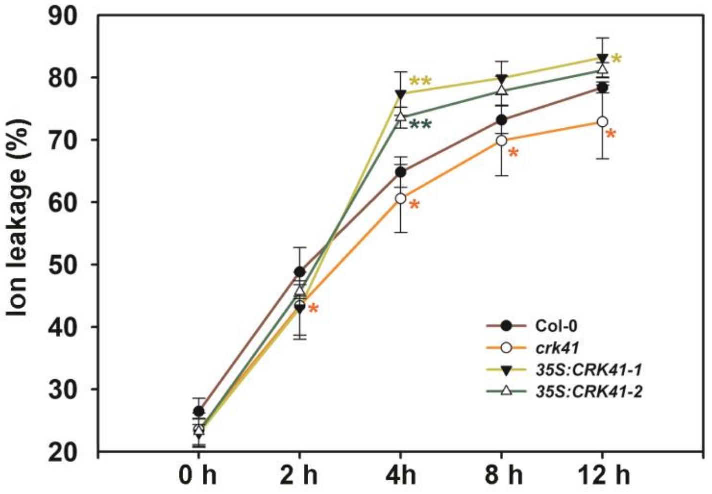

Figure S3. Ion leakage in seedlings of Col-0, *crk41* mutant, *35S:CRK41-1* and *35S:CRK41-2* treated with NaCl. SD is represented by the error bars. * $p < 0.05$; ** $p < 0.01$ vs. wild type in the same condition.

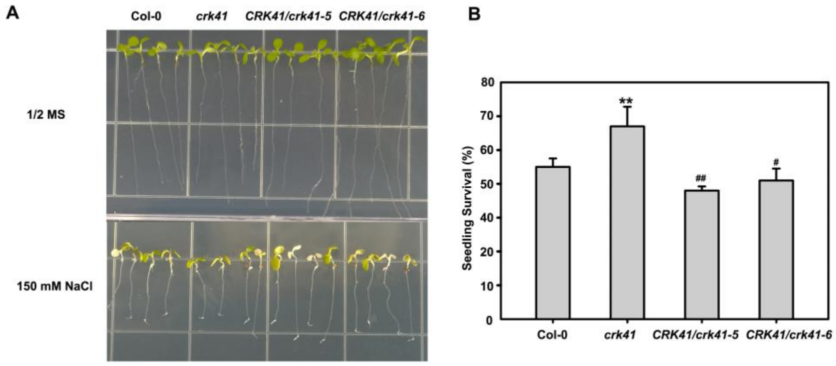

Figure S4. The salt-sensitive phenotype could be restored in the *crk41* complemented lines. (A) The growth phenotypes of the seedlings in Col-0, *crk41* mutant, *CRK41*/*crk41-5* and *CRK41*/*crk41-6* (6-day-old) after 150 mM NaCl treatments for 3 d.(B) The seedling survival rates of Col-0, *crk41* mutant, *CRK41*/*crk41-5* and *CRK41*/*crk41-6* (6-day-old) were measured after 150 mM NaCl treatment for 5 d. SD is represented by the error bars; $n = 3$ (at least 60 seeds were measured in per genotype of each replicate). ** $p < 0.01$ vs. wild type; # $p < 0.05$; ## $p < 0.01$ vs. *crk41* mutant.

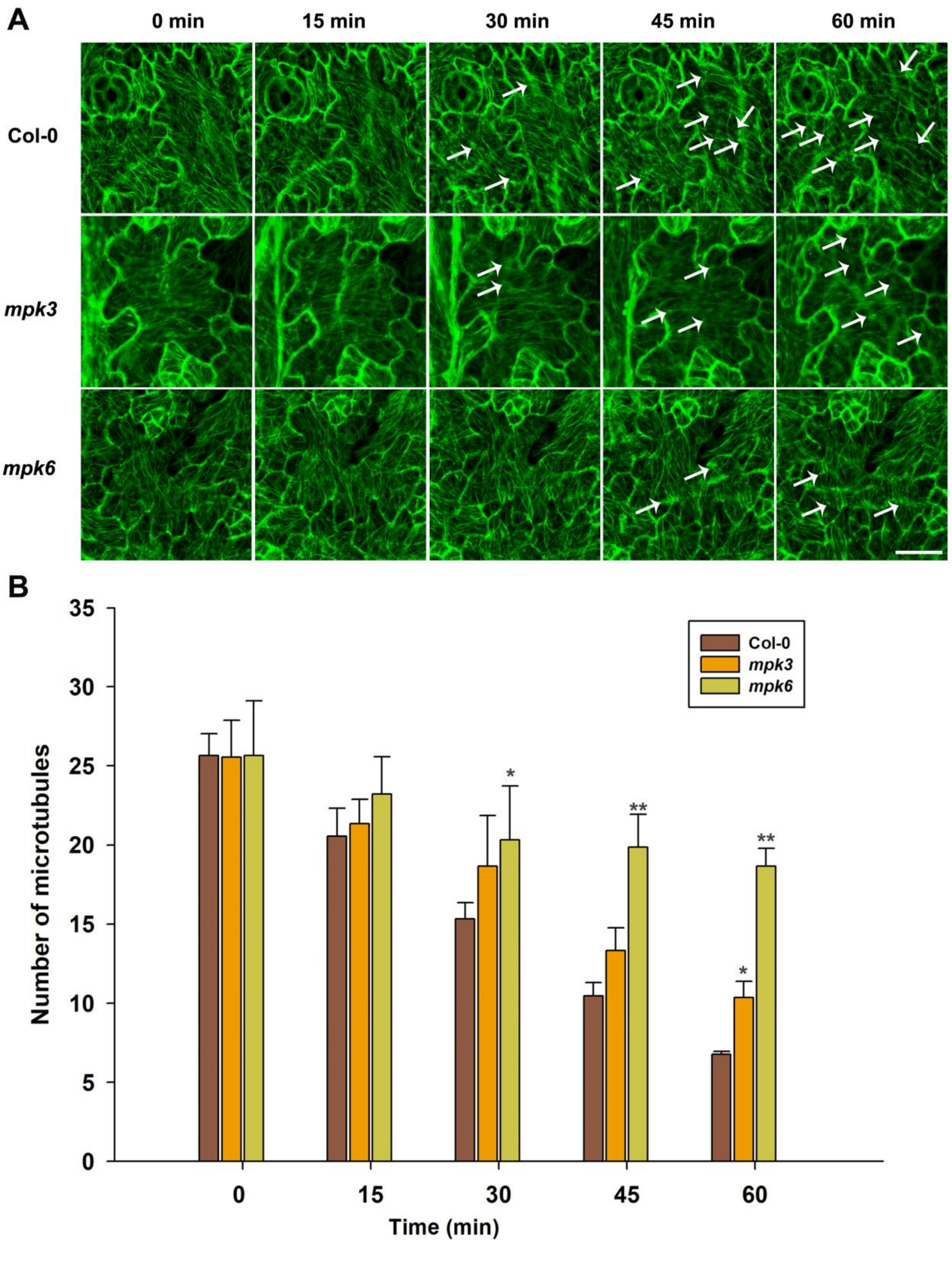

Figure S5. *MPK3* and *MKP6* have been identified as regulators of cortical microtubule depolymerization when exposed to salt stress. (A) Analysis of the cortical microtubule modifications caused by 150 mM NaCl in Col-0, *mpk3* and *mpk6* was conducted through a series of sequential images (scale bar =20 μm). The arrows indicated representative microtubule depolymerization.(B) Image tool software was utilized to quantify cortical microtubules in Col-0, *mpk3* and *mpk6*, with a minimum of 18 cells from three samples being analyzed. SD is represented by the error bars. * $p < 0.05$; ** $p < 0.01$ vs. wild type in the same condition.
